# Characterization of *Vibrio* spp. in environmental water samples collected from flood prone areas of Bangladesh and their antibiotic resistance profile

**DOI:** 10.3934/microbiol.2021028

**Published:** 2021-11-19

**Authors:** Md. Aoulad Hosen, Fozol Korim Ovi, Harunur Rashid, MD. Hasibul Hasan, Md. Abdul Khalek, Mahmudul Hasan, Farhana Easmin, Nazmi Ara Rumi, Mohammad Shariful Islam

**Affiliations:** 1 Department of Microbiology, Gono Bishwabidyalay, Dhaka, Bangladesh; 2 Sylhet Agricultural University, Sylhet, Bangladesh; 3 Department of Microbiology, Primeasia, Dhaka, Bangladesh; 4 Department of Microbiology, Hajee Mohammad Danesh Science & Technology University Dinajpur, Bangladesh; 5 Department of Microbiology, Jagannath University, Dhaka, Bangladesh

**Keywords:** surface water, underground water, *Vibrio cholera*, antibiotic sensitivity

## Abstract

Last cholera epidemic has been recorded in Bangladesh between 1992–1993, while few sporadic localized outbreaks have been reported as recent as 2005. Serotype O1 of *Vibrio cholera* is considered as the principal causative agent which transmits through contaminated drinking water resulting that epidemic. Therefore, the objective of this research was to isolate *V. cholera* in 3 different water sources; River, pond and tube-well, in 5 different locations of Gazipur, Bangladesh, and to analyze their antibiogram study. A total of 45 water samples were randomly collected for the isolation and identification of *Vibrio* spp. Samples are then serially diluted in alkaline peptone water and streak on Thiosulfate Citrate Bile Salt Sucrose-TCBS agar for quantification of *V*. spp. For *V. cholera* isolation water samples were first enriched in nutrient broth at 37 °C for 16 hours followed by cultivation in selective media; TCBS agar at 37 °C for 24 hours. Yellow colonies on TCBS agar were screed as *V. cholera* and was confirmed by analyzing their biochemical characteristics like Catalase, Oxidase, MR, VP, Indole, Sugar fermentation. Following isolation antibiotic sensitivity test was performed on each *V. cholera* isolates to determine their antibiotic sensitivity profile. The results showed, out of 45 samples 12 contained *V. cholera*. Tube-well water has significantly lower concentration (log CFU/mL) of *V*. spp. than river and pond water (P < 0.05). Bacterial concentration doesn't deviate (P > 0.05) significantly in 5 different location the sample was collected from. All the 12 isolates were sensitive to Gentamicin and ciprofloxacin (100%), while Chloramphenicol (91.67%), Sulfamethoxazole (91.67%), Azithromycin (66.67%) showed high sensitivity. Isolates showed marginal sensitivity towards Tetracycline (33.33%), and Cephalexin (16.67%) and 100% resistance against antibiotics like Vancomycin, Penicillin, Erythromycin, and Nalidixic Acid. Based on these data we recommend using tube-well water instead of river and pond water for drinking purposes. Furthermore, we suggest selective use of sensitive antimicrobials listed here for therapeutics of cholera outbreak.

## 1. Introduction

*V*. *cholera* is a gram-negative, facultative, motile anaerobe that secrets a diarrhoeagenic protein called cholera toxin [1]. The organism has over 200 serogroups, but only the O1 and O139 serogroups have been linked to the diarrheal disease commonly known as cholera [2]. In third world countries, the organism typically transmits by drinking contaminated surface water [3], whereas, in developed countries, transmissions are associated with raw or undercooked shellfish consumption [4]. Cholera is a frequent occurrence in Bangladesh, with seasonal outbreaks occurring annually [5]. Since *V. cholera* is predominantly an aquatic organism, the propagation and epidemiology of these outbreaks are highly influenced by contaminated water sources and flooding. In most rural areas of Bangladesh, access to potable clean drinking water is minimal, especially during annual flooding. A vast majority of the population still drinks untreated surface water in rural areas of the country and most of these annual outbreak buds in those populations.

Furthermore, underground water sources like tube wells are often submerged and contaminated by flood water during annual flooding. Thus, it is crucial to perform a comparative study of *V. cholera* contamination among different surface and underground water sources as a part of consistent surveillance operation. Besides, annual cholera outbreaks are often treated with the same group of antibiotics, resulting in a high antibiotic resistance in *V. cholera* strains against commonly used antibiotics. A yearly evaluation of the antibiotic resistance profile of field strains is necessary for the effective therapeutic use of available antibiotics. Therefore, this research aimed at quantifying *V*. spp. in different water sources of Bangladesh and evaluating the antibiotic resistance profile of *V. cholera* to estimate and mitigate the risk of the annual cholera outbreak.

## 2. Materials and methods

### 2.1. Sample collection, bacteria isolation, and quantification

**Figure 1.**
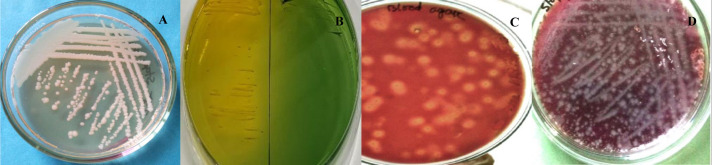
*V*. spp. on Nutrient's Agar (A), TCBS Agar left the yellow colony and green colony right (B), blood agar hemolytic colony (C), and non-hemolytic colony on blood agar (D).

A total of 45 environmental water samples were aseptically collected from pond, river and tube-well of different designated areas of Gazipur district, Bangladesh (Benupur, Chandabaha, Kaliakoir, Sutrapur and Begunbari). Following collection 4 samples are then serially diluted in alkaline peptone water and streak on selective media of Thiosulfate Citrate Bile Salts Sucrose (TCBS) agar, (Hi media, India) and incubated 37 °C for 24 hours. Following incubation, colonies with shiny yellow color and smooth, convex, and slightly flattened texture with opaque centers (Figure 1A, 1B) were used in viable count of *V*. spp. [6]. For bacteria isolation 1 mL of buffer peptone solution (1:10 dilution) was enriched in nutrient broth at 37 °C for 16 hours and then transferred in selective media (TCBS agar plate) for incubation (37 °C for 24 hours). Then one colony was randomly selected from each plate for biochemical analysis and hemolysis test (Figure 1C, 1D).

**Table 1.** Concentration (µg /disc) of antibiotic disc used for antimicrobial resistance test.

Antibiotics	Symbol	Disc concentration (µg /disc)
Ciprofloxacin	(CIP)	5
Gentamycin	(GEN)	10
Penicillin	(P)	10
Vancomycin	(VA)	30
Cephalexin	(CN)	30
Chloramphenicol	(C)	30
Tetracycline	(TE)	30
Erythromycin	(E)	15
Sulfamethoxazole	(SXT)	25
Nalidixic Acid	(NA)	30
Azithromycin	(AZ)	15

### 2.2. Biochemical test

*V*. spp. isolated in selective media were confirmed as *V. cholera* by different biochemical tests Catalase, Oxidase, MR, VP, Indole, glucose, maltose, mannitol and sucrose fermentation) according to the methodology described in [7].

**Table 2.** Bacterial concentration in different sources collected from 5 different locations.

Sample type	Bacterial conc. (Log CFU/mL)		
	10^−3^ DF^1^	10^−4^ DF^1^	10^−5^ DF^1^
River	4.96^A^	5.89^A^	6.79^a^
Pond	4.98^A^	5.92^A^	6.80^a^
Tube well	4.86^B^	5.73^B^	6.06^b^
SEM^2^	0.028	0.035	0.500
P value	<0.0001	<0.0001	<0.0001

### 2.3. Antibiotic susceptibility test

Antibiotic sensitivity test was performed according to Kriby-Bauer disc diffusion method [9] and following the guideline of Clinical and Laboratory Standards Institute [8]. A total of 11 commercially available antibiotics were used (Table 1) in this research to assess drug susceptibility and resistance of isolated species (Mast diagnostics Mersey side, UK). A single colony of pure culture isolated from the samples was incubated in nutrient broth at 37 °C for 16 hours. Then 0.1 ml of broth was spread on Mueller-Hinton agar plate using a cell spreader and an antibiotic disc was placed on top. The plates were then incubated in 37 °C for 24 hours. After incubation, the zone of inhibition near the discs was measured using a millimeter scale and categorized as resistant or sensitive according to the manufacturer's recommendation (Table 1).

**Table 3.** *V*. spp. concentration in water samples of 5 different location (Benupur, Chandabadha, Kaliakoir, Sutrapur and Begunbari).

Sample type	DF^1^	Bacterial concentration in each location (Log CFU/mL)				
		Benupur	Chandabaha	Kaliakoir	Sutrapur	Begunbari
River Burigonga	10^−3^	4.95	5.00	4.93	4.99	4.94
	10^−4^	5.88	5.93	5.87	5.90	5.88
	10^−5^	6.79	6.80	6.81	6.81	6.79
Pond	10^−3^	4.94	4.99	5.00	4.99	5.00
	10^−4^	5.90	5.89	5.93	5.90	5.87
	10^−5^	6.80	6.79	6.80	6.77	6.72
Tube-well	10^−3^	4.91	4.86	4.86	4.83	5.00
	10^−4^	5.81	5.76	5.71	5.68	5.89
	10^−5^	6.62	6.52	6.46	6.57	7.00

Average		5.84	5.83	5.82	5.83	5.90
SEM^2^		0.261	0.257	0.261	0.259	0.247

P-value		0.996

*Note: CFU= colony forming unite, ^1^DF = Dilution factor, ^2^SEM= Standard error of mean

### 2.4. Statistical analysis

A total of 5 replicate samples were randomly collected from each of the 5 different locations (Benupur, Chandabaha, Kaliakoir, Sutrapur, Begunbari) of each 3 water sources (river, pond and tube-well). Bacterial concentration in samples were log transformed and subjected to Shapiro-Wilk test for normality analysis. Bartlett's Test was performed to ensure the homogeneity of variance among the collected samples. The bacterial concentration in different water sources and at different locations were analyzed with one-way ANOVA using the GLM procedure of SAS software (version 9.2) under the following model. Yij = µ + Ti + δ_L_ + εij. Where, Yij = Bacterial concentration in each sample; µ = Overall mean bacterial concentration; Ti = Effect of water source; δ_L_ = Blocking effect of location and εij = random error. We assumed that the variation within the model, caused by from sampling location are normally distributed with a mean of 0 and a variance of σ_L_^2^. Random error εij of the model is also normally distributed with a mean of 0 and a variance of σ^2^. Both variances σ_L_^2^ and σ^2^ are independent of each other. For data analysis, P < 0.05 was considered statistically significant and when a significant difference is detected, the were subjected to the least significant difference test (LSD) for mean separation.

**Figure 2.**
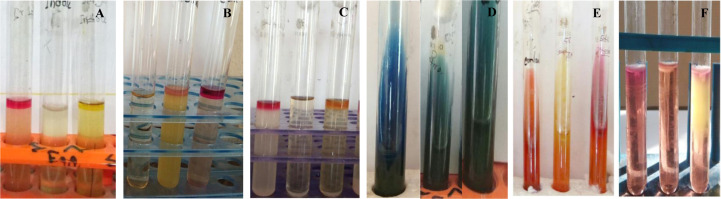
Biochemical test results. Indole test (A), Methyl red test (B), Voges-Proskauer test (C), Simmons citrate test (D), KIA test (E), and MIU test (F).

**Table 4.** Biochemical tests result of 12 isolated strains of *V. cholera*.

Tests	Results											
	R1	R3	R5	R6	R7	P1	P3	P5	P6	T3	T4	T7
Nit	+	+	+	+	+	+	+	+	+	+	+	+
Ox	+	+	+	+	+	+	-	+	+	+	-	+
Ind	+	+	+	+	-	+	+	+	+	+	+	+
Ci	+	+	+	+	+	+	+	+	+	+	+	+
MR	-	-	-	+	-	-	-	+	-	-	-	-
VP	+	+	+	+	+	+	+	+	+	+	+	+
MIU	+	+	+	+	+	+	+	-	+	+	+	+
Urease	-	-	-	-	-	-	+	-	-	-	-	-
KIA	Yb, Ys, G= -H2S= -	Yb, Ys, G= -H2S= -	Yb, Ys, G= -H2S= -	Yb, Ys, G= -H2S= -	Yb, Ys, G= -H2S= -	Yb Ys, G= -H2S= -	Yb, Ys, G= -H2S= -	Yb, Ys, G= -H2S= -	Yb, Ys, G= -H2S= -	Yb, Ys, G= -H2S= -	Yb, Ys, G= -H2S= -	Yb, Ys, G= -H2S= -
Glucose	+	+	+	-	+	+	-	+	+	+	-	+
Maltose	+	+	+	+	+	+	+	+	+	-	+	+
Mannitol	+	+	+	+	+	+	+	+	+	+	+	+
Sucrose	+	+	+	+	-	+	+	+	+	+	+	+
Gelatin hydrolysis	+	+	+	+	+	+	-	+	+	+	+	+

*Note: Legends: SL No.: Serial Number, Nit: Nitrate utilization test, Ox: Oxidase test, In: Indole test, Ci: Citrate test, MR: Methyl Red test, VP: Voges-Proskauer test, MIU: Motility indole urease, KIA: Kinglar iron agar +: positive, -: Negative, Y: Yellow, B: Butt, S: Slant, G: Gas.

## 3. Results and discussion

Viable counts were performed on TCBS agar plate which selects *V*. spp. based on their sucrose fermentation characteristics and the result is presented in Table 2. There was significantly higher *V*. spp. in pond and river water than tube-well water (P < 0.001) at all 3 dilution levels (dilution factor: 10^3^, 10^4^ and 10^5^). Bacterial concentration did not vary significantly based on the location of sample collection (P > 0.05) (Table 3).

**Table 5.** Comparative prevalence of *V*. *cholera* among the *V*. spp. isolated from different water sources.

Bacterial isolate	River water	Pond water	Tube well water	Total isolates	Percentage (%)
*V. cholera*	5 (41.67%)	4 (40%)	3(37.5%)	12	40
*V. parahimulyticus*	7 (58.33%)	6 (60%)	5 (62.5%)	18	60
Total isolates	12	10	8	30	

Total of 14 biochemical tests were performed on isolates of different samples and the result of those tests are presented in Table 4. Out of the 30 isolates, 12 isolates were positive in nitrate, oxidase, indole, citrate utilization, MR, motility, glucose, sucrose, mannitol, maltose, and gelatin hydrolysis agar test (Figure 2A, 2F). *V*. spp. were also found to be negative in MR, urease, and kingler iron agar test. Hemolytic characteristics of the isolates were also evaluated to differentiate between *V. cholera* and *V. parahaemolyticus* (Figure 1C, 1D). *V. cholera* are known to cause β-hemolysis whereas *V. parahaemolyticus* causes α-hemolysis. Based on this characteristic 12 of the initial isolates were classified as *V. cholera* and remaining 18 was classified as *V. parahaemolyticus*.

The comparative prevalence of *V. cholera* among the *V*. spp. isolated from different water sources are presented in Table 5. River water had the highest prevalence of *V. cholera* (5 out of 12 isolates; 41.67%) whereas, tube-well water had the lowest prevalence (3 out of 8; 37.5%).

**Table 6.** Outcome of antibiotic sensitivity test of 12 *V. cholera* isolates obtained from different water samples.

Isolate	GEN	CIP	CN	VA	P	C	TE	E	NA	AZ	SXT
R1	S	S	S	R	R	S	S	I	I	S	S
R3	S	S	S	R	R	S	S	R	R	S	S
R5	S	S	S	I	R	S	I	R	R	S	S
R6	S	S	S	R	R	S	I	I	R	R	S
R7	S	S	S	R	R	R	R	R	R	S	S
P1	S	S	S	R	R	S	R	R	R	S	S
P3	S	S	S	R	R	S	R	R	R	S	M
P5	S	S	S	R	R	I	I	R	R	S	S
P6	S	S	I	I	I	S	I	R	R	R	S
T3	S	S	I	R	R	S	S	I	R	S	S
T4	S	S	R	R	R	S	S	I	R	S	S
T7	S	S	R	I	R	S	I	R	R	S	S

*Note: GEN: Gentamycin, CIP: Ciprofloxacin, CN: Cephalexin, VA: Vancomycin, P: Penicillin, C: Chloramphenicol, TE: Tetracycline, E: Erythromycin, NA: Nalidixic Acid, AZ: Azithromycin, SXT: Sulfamethoxazole, R: River, P; Pond; T; Tap, s: Sensitive and r: Resistance. R_1,_ R_3,_ R_5,_ R_6,_ R_7_ are the *V. cholera* isolates collected from rivers, P_1_, P_3_, P_5_ and P_6_ are the *V. cholera* isolates collected from pond, T_3_, T_4_ and T_7_ are the *V. cholera* isolates collected from tube-well.

The results of antibiotic sensitivity test performed on 12 *V. cholera* isolates are presented in Table 6. The antibiotic sensitivity profiles of those isolates have been compiled in Table 7. All 12 isolates showed 100% sensitivity toward Gentamicin and ciprofloxacin. All the isolates showed multidrug resistance (Table 6). However, these isolates were susceptible to Chloramphenicol (91.67%), and Sulfamethoxazole (91.67%). Azithromycin (66.67%). Tetracycline (33.33%), and Cephalexin (16.67%) had moderate to low sensitivity. All 12 isolates showed 100% resistance toward Penicillin, Vancomycin, Erythromycin, and Nalidixic Acid. This result is congruent with the study of [10] performed in neighboring country Nepal, where they found their isolates sensitive to Ciprofloxacin, Ampicillin, and resistant to Nalidixic acid. However, unlike this study, their isolates also showed higher sensitivity toward Erythromycin and Tetracycline. The majority of resistance in environmental species are thought to have originated from historically resistant organisms. As a result, it's essential to keep track of both the frequency and the antimicrobial resistance profile of *V. cholera* to identify the high-risk water sources. To minimize the risk of cholera transmission through contaminated water, we recommend screening various water sources against this pathogenic bacteria before using it for washing, drinking and irrigation. Vulnerable populations, especially farmers in rural areas, should take appropriate precautions to avoid cholera transmission through water [11]. There were some limitations to our research. Due to the funding constrain, a limited number of samples were collected, which might not be sufficient to draw a precise conclusion. Our analysis still lacks molecular characterization of the isolates, which might have strengthened our conclusion.

**Table 7.** Antibiotic sensitivity profile of 12 isolates *V*. spp. obtained from different water samples.

Organism	Antibiotics	Susceptibility (%)	Resistance (%)
*V. cholera*	Gentamycin (GEN)	12(100%)	0(0%)
	Ciprofloxacin (CIP)	12(100%)	0(0%)
	Cephalexin (CN)	2(16.67%)	10(83.33%)
	Vancomycin (VA)	0(0%)	12(100%)
	Penicillin (P)	0(0%)	12(100%)
	Chloramphenicol (C)	11(91.67%)	1(8.33%)
	Tetracycline (TE)	4(33.33%)	8(66.67%)
	Erythromycin (E)	0(0%)	12(100%)
	Sulfamethoxazole (SXT)	11(91.67%)	1(8.33%)
	Nalidixic Acid (NA)	0(0%)	12(100%)
	Azithromycin (AZ)	8(66.67%)	4(33.33%)

## 4. Conclusion

Based on the data of our experiment we conclude that *V. cholera* is endemic to the surface water sources like pond and river in Gazipur region of Bangladesh. Underground water like tube well has comparatively lower concentration of *V. cholera* Antibiotics like, Gentamicin, Ciprofloxacin, Chloramphenicol, Sulfamethoxazole and Azithromycin are highly effective against the *V. cholera* isolates collected in this study. We suggest the application of these antibiotics in therapeutics of annual cholera outbreak. Furthermore, we highly recommend prioritizing underground water over surface water as drinking water source.
